# MOBIDERM® Autofit as a Novel Approach for Saphenous Vein Harvest Site Complications: Therapeutic and Prophylactic Applications in Lymphatic Disturbance

**DOI:** 10.7759/cureus.93205

**Published:** 2025-09-25

**Authors:** Yukio Umeda, Kumi Wakida, Ayako Tosaki, Kazuhiro Nishida, Shohei Mitta, Yukihiro Matsuno, Shoji Yoshikawa, Kenichiro Azuma

**Affiliations:** 1 Department of Cardiovascular and Thoracic Surgery, Gifu Prefectural General Medical Center, Gifu, JPN; 2 Department of Nursing, Gifu Prefectural General Medical Center, Gifu, JPN; 3 Department of Food and Nutritional Science, Toita Women’s College, Tokyo, JPN

**Keywords:** compression therapy, coronary artery bypass grafting, lower limb lymphedema, lymphatic disturbance, mobiderm® autofit, saphenous vein harvest, wound complication

## Abstract

Saphenous vein harvest site complications remain a concern after coronary artery bypass grafting, particularly when lymphatic disturbance contributes to delayed healing. We describe two cases in which MOBIDERM^®^ Autofit (Thuasne, Levallois-Perret, France), a micropad-based compression device originally developed for lymphedema management, was applied either therapeutically in a patient with wound infection and persistent edema or prophylactically in a high-risk patient with early postoperative swelling. In both situations, its use was associated with favorable healing and uncomplicated recovery, suggesting that this device may offer a practical adjunctive option for preventing and managing postoperative harvest site complications.

## Introduction

Saphenous vein (SV) harvest site complications remain clinically relevant after coronary artery bypass grafting (CABG), as they can delay mobilization, prolong hospitalization, increase costs, and impair long-term quality of life [1]. Although endoscopic vein harvesting (EVH) has reduced the incidence of wound problems [2], the recent reappraisal of the no-touch technique to improve long-term graft patency has reintroduced the challenge of managing harvest site complications [3-5].

Importantly, these complications are not only a surgical issue but also a multidisciplinary concern involving cardiologists, internists, wound care specialists, and rehabilitation teams. Management strategies, therefore, extend beyond the operative field and include systemic measures such as infection control, nutritional optimization, and early mobilization, in addition to local wound management.

Well-recognized risk factors for SV harvest site complications include diabetes, obesity, peripheral arterial disease, hypoalbuminemia, steroid use, and smoking [2,6,7]. In addition, disturbance of lymphatic drainage and preoperative venous hypertension have been reported as important contributors [6,8]. Although these factors have been reported as important contributors, their prevalence among CABG patients remains unclear and is not systematically described in the literature. Conventional preventive measures, such as drains, negative pressure wound therapy (NPWT), limb elevation, and elastic bandages, are widely applied and generally effective [7,9,10]. Nevertheless, persistent edema, prolonged exudation, and delayed healing remain challenging in selected cases. Moreover, elastic bandages can sometimes exacerbate lymphatic impairment, causing blistering and patient discomfort.

Advances in lymphedema management have introduced compression devices designed to improve lymphatic flow. MOBIDERM^®^ Autofit (Thuasne, Levallois-Perret, France), consisting of foam micropads that generate differential pressure zones, has demonstrated efficacy in breast cancer-related lymphedema and venous ulcer care [11-14]. However, its role in SV harvest site complications has not been previously described.

Here, we report two patients in whom MOBIDERM^®^ Autofit was used as an adjunct to conventional management: one therapeutically for wound infection and severe edema, and one prophylactically in a high-risk patient with early postoperative swelling. These cases highlight a potential application of a lymphedema-specific device in CABG patients, but they should be regarded as preliminary and hypothesis-generating observations.

## Case presentation

Case 1: therapeutic use

Patient Profile

A 74-year-old man with hypertension, hyperlipidemia, chronic kidney disease, chronic obstructive pulmonary disease, cerebrovascular disease, and a prior abdominal aortic graft replacement was referred after a routine echocardiography revealed left ventricular wall motion abnormality. Coronary angiography showed 75% stenosis of the left main trunk. He had quit smoking three years earlier. Duplex ultrasonography was performed for graft assessment, which showed adequate saphenous vein quality and no evidence of preoperative venous hypertension or lymphedema. Preoperative evaluation demonstrated mild renal impairment, hypoalbuminemia, and moderate surgical risk (JAPAN SCORE II: 4.5%, STS: 1.54%).

Operative Procedure

Under cardiopulmonary bypass and cardioplegic arrest, CABG with two grafts was performed (LITA to #8, Ao-SVG to #14). The left SV was harvested from the lower leg, but the patient had notably fragile skin with minimal subcutaneous tissue. As subcutaneous buried sutures were not feasible, the wound was closed with interrupted sutures with a closed suction drainage (SB^TM^ vac, Sumitomo Bakelite Co., Ltd., Tokyo, Japan). An occlusive hydrocolloid dressing (Karayahesive^®^, ALCARE Co., Ltd., Tokyo, Japan) was applied to the entire wound.

Postoperative Course

The patient was extubated on postoperative day (POD) 1 and discharged from the ICU on POD 3. coronary CT on POD 5 confirmed patency of all grafts. The sternal wound healed uneventfully, but the leg wound developed complications.

Early course: Drain removal was performed on POD 3. Despite consistent instructions for limb elevation during the bedridden period to minimize edema, these findings persisted. Sutures were removed on POD 13, but wound dehiscence measuring approximately 1 cm in width and 15 cm in length developed, accompanied by purulent discharge from the wound. Wound culture grew MRSA and Pseudomonas aeruginosa, for which treatment with ceftazidime and teicoplanin was initiated. After two weeks of antibiotic therapy, the infection was successfully controlled. Surgical debridement was performed by the plastic surgery team, and the debridement defect measured approximately 5 × 30 cm. Local wound management with basic fibroblast growth factor spray (Fiblast®; KAKEN Pharmaceutical Co., Ltd., Tokyo, Japan) and prostaglandin ointment (Prostandin® ointment; ONO Pharmaceutical Co., Ltd., Osaka, Japan) was continued.

Renal function: Persistent hypoalbuminemia and poor oral intake contributed to fluid imbalance. Renal function gradually deteriorated, necessitating initiation of dialysis on POD 44. Dialysis was successfully discontinued on POD 56 as fluid balance improved, but leg edema and high-volume exudation persisted.

Discharge and readmission: Due to the patient’s strong wish for discharge, he was discharged on POD 63 with ongoing home wound care. However, worsening renal function prompted readmission on POD 71, and dialysis was restarted.

Advanced Wound Management

**Figure 1:**
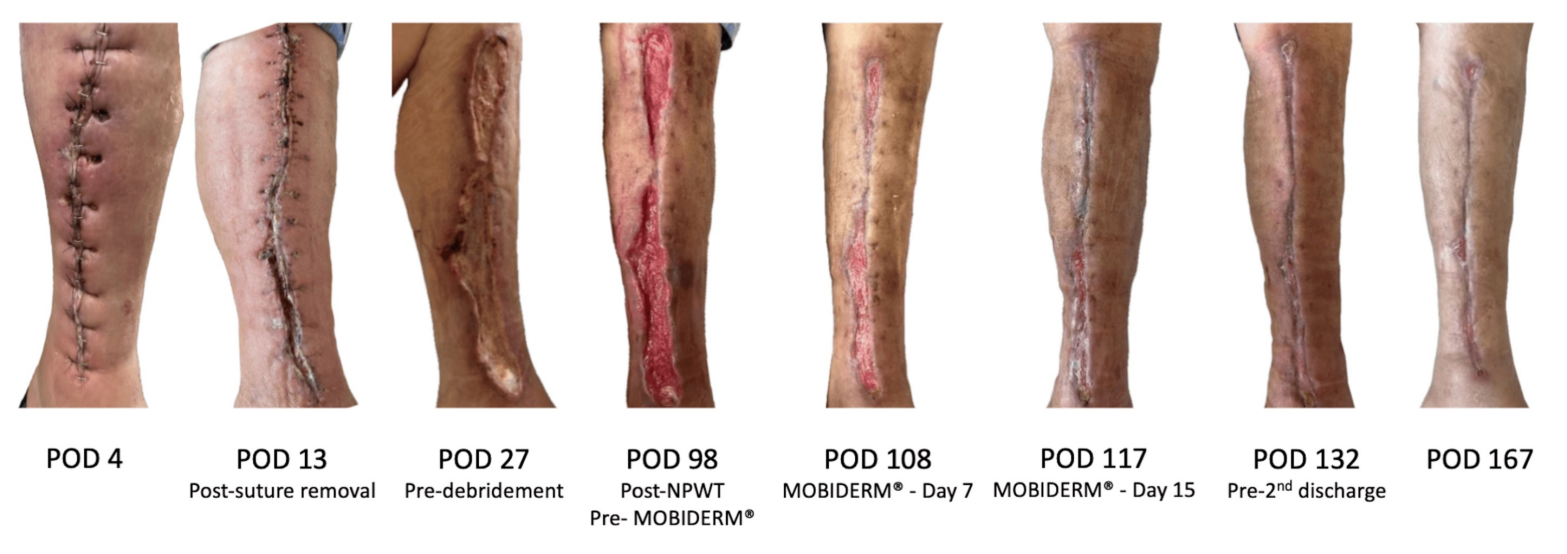
Therapeutic application of MOBIDERM® Autofit for lymphatic disturbance-related delayed healing (Case 1) On POD 4, the left saphenous vein (SV) harvest site showed epidermal loss lateral to the sutures, swelling, and erythema of the entire lower leg. Following suture removal on POD 13, wound dehiscence developed in the lower half of the incision. Despite continuous wound management, healing was delayed, and dehiscence extended to involve the entire wound, with necrotic tissue present on the wound surface (POD 27). Even after the completion of NPWT on POD 98, leg edema was reduced; however, marked continuous serous exudation persisted and hindered wound healing. The subsequent introduction of MOBIDERM^®^ Autofit (Thuasne, Levallois-Perret, France) led to a rapid reduction in both edema and exudate, thereby markedly promoting wound repair (POD 108–117). By POD 167, the saphenous vein harvest site had achieved complete epithelialization with stable healing. NPWT: negative pressure wound therapy; POD: postoperative day

The plastic surgery team initiated NPWT (RENASYS TOUCH; Smith & Nephew plc, London, United Kingdom) between postoperative days (POD) 76 and 97. Although repeated debridement and NPWT promoted exposure of healthy granulation tissue and reduced edema in the absence of infection, marked continuous serous exudation resulted in delayed epithelialization and only minimal wound contraction. As lymphatic disturbance was considered the main cause, a micropad compression device (MOBIDERM^®^ Autofit) was applied from POD 101. The device, applied from the knee to the ankle and worn continuously except during wound care, generated an estimated compression pressure of 20-30 mmHg through alternating high- and low-pressure zones created by the foam micropads. Following its introduction, the leg edema rapidly subsided, and wound healing progressed significantly, with both edema and exudate almost completely resolving within 5 days. By POD 120, the patient was switched to elastic tubular compression stockings (Lava Lava 2; Kyushu Medical Service, Tokyo, Japan). On POD 134, he was transferred to a rehabilitation facility with stable wound healing and improved mobility, as illustrated in Figure 1, showing the wound healing process, including post-discharge at the SV harvest site. The corresponding serial laboratory parameters related to wound healing, aligned with the clinical course shown in Figure 1, are presented in Table 1.

**Table 1:** Serial laboratory parameters related to wound healing (Case 1) Values represent sequential changes in laboratory markers associated with wound healing, aligned with the timeline of sequential wound images and clinical events illustrated in Figure 1. Reference ranges may vary depending on laboratory standards. POD: postoperative day

Parameters	Preoperative value	POD 4	POD 13	POD 27	POD 98	POD 108	POD 117	POD 132	POD 167	Reference Range	Unit
Total Protein	6.3	5.1	5.9	5.9	5.2	5.4	5.1	5.6	5.8	6.6–8.1	g/dL
Albumin	3.8	2.8	3.1	3.2	2.2	2.2	2.1	2.3	2.0	4.1–5.1	g/dL
Blood Urea Nitrogen	28	51	47	40	47	26	26	29	50	8–20	mg/dL
Creatinine	1.45	2.05	2.4	2.00	6.04	4.07	4.41	3.89	2.63	0.67-1.07	mg/dL
Estimated Glomerular Filtration Rate	37.57	25.72	21.65	26.42	7.89	12.15	11.13	12.76	19.58		mL/min/1.73 m²
Red Blood Cell Count	393	341	373	388	270	291	275	290	287	435-555	×10⁴/μl
Hemoglobin	14.0	11.4	12.1	13.2	8.9	10.0	9.4	9.7	9.8	13.7-16.8	g/dL

Case 2: prophylactic use

Patient Profile

A 52-year-old man with hypertension, hyperlipidemia, and rheumatoid arthritis on methotrexate presented with unstable angina and a recent myocardial infarction. Coronary angiography showed multivessel disease (#4PD 75%, #5 50%, #6 100%, #13 90%). Duplex ultrasonography confirmed suitable saphenous vein segments with no venous hypertension or lymphedema. Preoperative risk was low to moderate (JAPAN SCORE II: 0.6%, STS: 0.5%).

Operative Procedure

An intra-aortic balloon pump (IABP) was inserted via the right femoral artery before urgent CABG. Although the left internal thoracic artery and the left great saphenous vein were initially intended as grafts, the harvested internal thoracic artery demonstrated insufficient free flow. Therefore, an additional saphenous vein was harvested from the right lower leg.

Under cardiopulmonary bypass and cardioplegic arrest, aorto-coronary bypass grafting was performed (Ao-SVG to #8 and Ao-SVG to #12-#4PD). Considering the patient’s obesity (BMI 31%) and chronic immunosuppressive therapy, NPWT using 3M^TM^Prevena^TM^ (Solventum Corp., St. Paul, Minnesota, United States) was applied to the median sternotomy wound, and closed suction drainage systems (SB^TM^ vac) were placed at both SV harvest sites.

Postoperative Course

The IABP and wound drains were removed on postoperative day POD 1, and the patient was extubated and discharged from the ICU on POD 2. Coronary CT performed on POD 6 confirmed patency of all grafts.

Wound management proceeded as follows: NPWT was discontinued on POD 7, with the sternotomy site showing satisfactory healing. In contrast, by POD 4, the left lower leg exhibited marked edema and exudate. We applied MOBIDERM^®^ Autofit and continued it until POD 11. Following the application of MOBIDERM^®^, the calf circumference measured 10 cm distal to the inferior pole of the patella decreased from 42.6 cm to 37.0 cm, showing a reduction of 5.6 cm.

**Figure 2:**
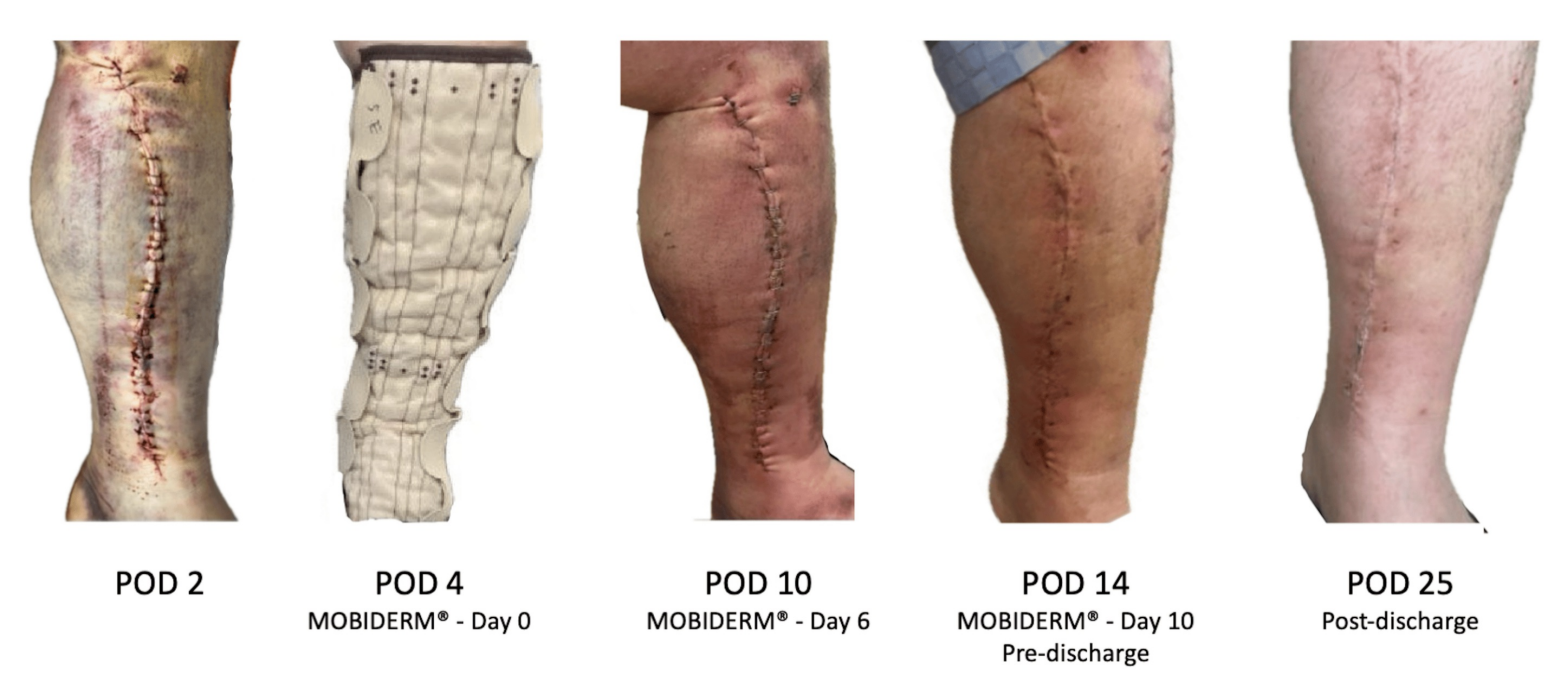
Prophylactic application of MOBIDERM® Autofit in a high-risk case for delayed wound healing (Case 2) At the immediate postoperative period, the left saphenous vein harvest site showed extensive subcutaneous hemorrhage, marked edema, and exudation. Given the patient’s obesity (BMI 31%) and chronic immunosuppressive therapy, he was considered at high risk of delayed wound healing. Therefore, MOBIDERM^®^ Autofit (Thuasne, Levallois-Perret, France) was applied prophylactically from POD 4 for 10 days, resulting in a marked reduction of edema and exudate. The harvest site subsequently demonstrated satisfactory wound healing. POD: postoperative day

The patient was discharged on POD 15 with elastic compression stockings (K tube^TM^, Batel Plus Corp., Tokyo, Japan) in place. At the outpatient follow-up visit on POD 25, the SV harvest site where MOBIDERM^®^ Autofit had been applied demonstrated favorable healing, as illustrated in Figure 2. The corresponding serial laboratory parameters related to wound healing, aligned with the clinical course shown in Figure 2, are presented in Table 2.

**Table 2:** Serial laboratory parameters related to wound healing (Case 2) Values represent sequential changes in laboratory markers associated with wound healing, aligned with the timeline of sequential wound images and clinical events illustrated in Figure 2. Reference ranges may vary depending on laboratory standards. POD: postoperative day

Parameters	Preoperative value	POD 2	POD 4	POD 10	POD 14	POD 25	Reference Range	Unit
Total Protein	7.3	5.0	5.4	6.0	5.6	6.9	6.6–8.1	g/dL
Albumin	4.2	2.6	2.8	2.8	2.7	3.3	4.1–5.1	g/dL
Blood Urea Nitrogen	9	7	12	11	10	9	8–20	mg/dL
Creatinine	0.80	0.54	0.69	0.79	0.82	0.83	0.67-1.07	mg/dL
Estimated Glomerular Filtration Rate	79.68	122.48	93.67	80.78	77.55	76.53		mL/min/1.73 m²
Red Blood Cell Count	489	329	301	333	337	423	435-555	×10⁴/μl
Hemoglobin	15.8	10.1	9.3	10.3	10.4	12.6	13.7-16.8	g/dL

In summary, conventional management, such as limb elevation and compression, was initially employed in both patients, and MOBIDERM^®^ Autofit was introduced as an adjunct when delayed healing or high risk of lymphatic disturbance was encountered.

## Discussion

The superiority of the internal thoracic artery (ITA) in terms of long-term patency and its beneficial effect on survival has been well established in CABG [15]. However, harvesting bilateral ITAs is known to compromise sternal blood flow and increase the risk of mediastinitis in certain patient populations [16,17]. Therefore, in real-world clinical practice, the need for SV grafts (SVGs) remains high [1].

The principal problem associated with SV harvesting is delayed wound healing at the harvest site. Complications such as prolonged bleeding, persistent exudation, and infection can delay recovery, prolong hospital stay, increase postoperative discomfort, impair mobility, and in some cases necessitate additional interventions or readmission. Importantly, the clinical consequences of SV harvest site complications are not confined to the early postoperative period. Long-term sequelae have also been recognized, most notably chronic post-surgical pain (CPSP) [18]. The incidence of CPSP following CABG is reported to exceed 20%, with the highest prevalence observed at SV harvest sites. CPSP is frequently associated with neuropathic pain symptoms and exerts a substantial impact on instrumental activities of daily living. In fact, a case has been reported in which, 11 years after CABG, the patient developed lymphedema at the SV harvest site accompanied by recurrent cellulitis, severe pain, sleep disturbance, and markedly reduced quality of life [6]. In recent years, the advent of no-touch harvesting techniques has demonstrated improved graft outcomes [3,4], and this has drawn increasing attention to the importance of optimal wound healing at the SV harvest site [5,19].

Factors reported to contribute to delayed healing at the SV harvest site include female gender, diabetes mellitus, obesity, hypoalbuminemia, anemia, peripheral vascular disease, steroid use as preoperative factors, and venous stasis and lymphatic disturbance as harvesting-related factors [2,6]. Large lymphatic vessels lie adjacent to the saphenous vein and are likely to be injured during vein harvesting [8]. Indeed, there have been reports focusing on this aspect, such as evaluations of lymphatic factors in conventional open versus endoscopic vein harvesting (EVH), and the use of NPWT at the harvest site [7,9].

However, preventive strategies for delayed healing have not been standardized. Conventional measures have included techniques for harvesting and skin closure, the use of drains, application of NPWT, and elastic bandages serving both for hemostasis and wound management [2,7,10]. Despite employing such strategies in combination, we frequently encountered complications such as prolonged exudation, wound infection, and dehiscence. Notably, in cases where elastic bandages were applied with inappropriate tension, blister formation was observed, which was considered to potentially impede lymphatic flow. Postoperative compression might be clearly beneficial for hemostasis to reduce hematoma formation and blood loss, and to reduce edema to control pain and wound healing. However, excessive compression can result in nerve palsy, blistering, pressure sore development, and even necrosis [20]. 

MOBIDERM^®^ Autofit generates alternating high- and low-pressure zones through its micropad structure, enhancing lymphatic drainage and collateral pathway formation. This differential pressure also softens fibrotic tissue, thereby improving compliance and reducing lymphatic resistance.

Clinical evidence supports these mechanistic insights. In a case series of patients with stage II breast cancer‑related lymphedema undergoing complete decongestive therapy, application of MOBIDERM^®^ Autofit during the intensive phase resulted in a mean lymphedema volume reduction of approximately 36.7%, without reported adverse events. The device was also rated highly in terms of comfort and ease of use by both patients and physicians [11]. Furthermore, in the MARILYN pilot randomized controlled trial evaluating MOBIDERM^®^ Autofit during the maintenance phase (specifically nocturnal usage) of lymphedema management, the device helped stabilize limb volume and attenuate functional symptoms compared with daytime compression alone. Compliance was high (approximately 85% of nights worn), and patients reported improved function and high satisfaction rates [12].

Another important advantage of MOBIDERM^®^ Autofit is its ease of use and applicability in everyday practice. Because it is designed for self-management, patients can achieve continuous and reproducible compression therapy outside the hospital setting, which enhances adherence and prevents recurrent lymphatic fluid accumulation. Taken together, these properties suggest that MOBIDERM^®^ Autofit not only alleviates lymphedema but also helps prevent its chronic progression, providing both functional and psychological benefits for affected patients [13,14].

Based on these considerations, we applied MOBIDERM^®^ Autofit, which has been shown to be effective in improving lymphatic disturbance, in two cases: one as a therapeutic measure for an SV harvest site with infection and dehiscence, and another as a prophylactic measure to prevent delayed healing in a case with severe edema. Favorable outcomes were achieved in both cases.

Conventional management, including layered bandages, elevation, and compression stockings, remains the mainstay of prophylaxis and treatment for SV harvest site complications. These methods are cost-effective and adequate in most patients. Our report does not challenge this standard; rather, it illustrates that in selected high-risk or refractory cases, supplementary measures such as MOBIDERM^®^ Autofit may provide additional benefit.

From a practical perspective, the availability and reimbursement status of compression devices such as MOBIDERM^®^ Autofit vary between healthcare systems [21-25]. In countries where lymphedema management devices are covered by public or private insurance, early adoption may be facilitated, whereas in settings without reimbursement, the financial burden on patients could limit their use. Therefore, in addition to clinical validation, future studies should also address cost-effectiveness and healthcare policy considerations to support broader implementation in postoperative wound care.

Nevertheless, this report is limited to two observations, and further evidence is required to validate the clinical role of MOBIDERM^®^ Autofit in this setting. In particular, prospective studies incorporating standardized wound assessment tools, such as the ASEPSIS (Additional treatment, Serous discharge, Erythema, Purulent exudate, Separation of deep tissues, Isolation of bacteria, and Stay) [26], would help to determine whether early detection of wound complications should trigger the initiation of MOBIDERM^®^ therapy. Moreover, analyses of preoperative risk factors are needed to identify patient populations most likely to benefit from its prophylactic use. Taken together, these cases should be regarded as observational experiences that provide preliminary, hypothesis-generating insights. Given the heterogeneity of wound healing complications, the outcomes reported here cannot be generalized, but rather illustrate a potential therapeutic concept that warrants systematic validation.

## Conclusions

SV harvest site complications remain a clinically significant issue after CABG, with consequences that extend beyond the immediate postoperative period. Despite advances in harvesting techniques and preventive strategies, lymphatic disturbance continues to pose challenges for wound healing.

Our two cases suggest that MOBIDERM^®^ Autofit may be considered as a supplementary option for managing lymphatic disturbance-related complications at SV harvest sites. However, conventional measures remain first-line, and our observations are purely anecdotal. No definitive conclusions can be drawn from such limited data. Larger prospective studies are needed to validate whether this device improves outcomes beyond standard compression therapy.
